# Transcriptional dynamics and regulatory function of milRNAs in *Ascosphaera apis* invading *Apis mellifera* larvae

**DOI:** 10.3389/fmicb.2024.1355035

**Published:** 2024-04-08

**Authors:** Xiaoxue Fan, Xuze Gao, He Zang, Zhitan Liu, Xin Jing, Xiaoyu Liu, Sijia Guo, Haibin Jiang, Ying Wu, Zhijian Huang, Dafu Chen, Rui Guo

**Affiliations:** ^1^College of Bee Science and Biomedicine, Fujian Agriculture and Forestry University, Fuzhou, China; ^2^Jilin Apicultural Research Institute, Jilin, China; ^3^Animal Husbandry Terminus of Sichuan Provincial Department of Agriculture and Rural Affairs, Chengdu, China; ^4^National and Local United Engineering Laboratory of Natural Biotoxin, Fuzhou, China; ^5^Apitherapy Research Institute of Fujian Province, Fuzhou, China

**Keywords:** *Ascosphaera apis*, chalkbrood, *Apis mellifera*, larva, milRNA, regulation, infection mechanism

## Abstract

In the present study, small RNA (sRNA) data from *Ascosphaera apis* were filtered from sRNA-seq datasets from the gut tissues of *A. apis*-infected *Apis mellifera ligustica* worker larvae, which were combined with the previously gained sRNA-seq data from *A. apis* spores to screen differentially expressed milRNAs (DEmilRNAs), followed by trend analysis and investigation of the DEmilRNAs in relation to significant trends. Additionally, the interactions between the DEmilRNAs and their target mRNAs were verified using a dual-luciferase reporter assay. In total, 974 *A. apis* milRNAs were identified. The first base of these milRNAs was biased toward U. The expression of six milRNAs was confirmed by stem–loop RT-PCR, and the sequences of milR-3245-y and milR-10285-y were validated using Sanger sequencing. These miRNAs grouped into four significant trends, with the target mRNAs of DEmilRNAs involving 42 GO terms and 120 KEGG pathways, such as the fungal-type cell wall and biosynthesis of secondary metabolites. Further investigation demonstrated that 299 DEmilRNAs (novel-m0011-3p, milR-10048-y, bantam-y, etc.) potentially targeted nine genes encoding secondary metabolite-associated enzymes, while 258 (milR-25-y, milR-14-y, milR-932-x, etc.) and 419 (milR-4561-y, milR-10125-y, let-7-x, etc.) DEmilRNAs putatively targeted virulence factor-encoded genes and nine genes involved in the MAPK signaling pathway, respectively. Additionally, the interaction between *ADM-B* and milR-6882-x, as well as between *PKIA* and milR-7009-x were verified. Together, these results not only offer a basis for clarifying the mechanisms underlying DEmilRNA-regulated pathogenesis of *A. apis* and a novel insight into the interaction between *A. apis* and honey bee larvae, but also provide candidate DEmilRNA–gene axis for further investigation.

## 1 Introduction

*Ascosphaera apis* (Ascomycota, Eurotiomycetes, Ascosphaerales), a fungal pathogen closely adapted to honey bees, exclusively infects bee broods and gives rise to chalkbrood disease, which severely damages the apicultural industry by causing a sharp reduction in colony population and productivity (Aronstein et al., 2010). Recent evidence demonstrates that *A. apis* is also infectious for adult bees (Maxfield-Taylor et al., 2015). *A. apis* spores cannot germinate on the larval cuticle; therefore, the infection in honey bee larvae starts when the fungal spores are ingested with food during their feeding (Aronstein et al., 2010). After being ingested, the *A. apis* spores start to germinate under a high CO_2_ concentration in the larval gut of the bees. These spores then become swollen and create germ tubes that grow into dichotomous mycelia, which penetrate the peritrophic membrane around the larval gut and gut wall into the body cavity, facilitated by a collaboration of mechanical force and enzyme action, followed by vegetative growth extending from the posterior end to the anterior end of the larva. Gradually, the entire larva becomes covered with a thick layer of white mycelium, after which fruiting bodies with new ascospores are formed on the aerial hyphae outside the dead larvae (Heath and Gaze, 1987). In the case of the presence of both mating types, spore cysts (ascomata) begin being generated. Ascospores, the only infective units causing chalkbrood (Wang, 2014), are formed in spore balls and located in resistant cysts (Liang et al., 2000). The spores contain two nuclei, the bigger one of which lies in the center, while the smaller one is situated near the end of the spore (Aronstein et al., 2010). The three-layered spore wall is tough, containing chitin as its major component, which helps the ascospores stay viable for many years (Vilcinskas, 2010). There is some indication that this fungus, which is one of the most contagious and destructive bee brood pathogens (Li et al., 2012), has been increasing in occurrence (Wang, 2014). The incidence of chalkbrood has been suggested to have been increasing for many years due to human activities (Evison, 2015). At present, effective and environmentally friendly therapeutic compounds are unavailable; however, some alternative strategies, such as management and sanitation, have been developed to fight against chalkbrood disease (Aynalem et al., 2022; Krutmuang et al., 2022).

MicroRNAs (miRNAs) are a class of small non-coding RNA (ncRNA) molecules that are approximately 22 ~ 25 nucleotides (nt) in length, which can partially or entirely bind to the 5’ UTR, 3’ UTR, or CDS of target genes to down- or up-regulate the expression of target genes (Bushati and Cohen, 2007; Fabian and Sonenberg, 2012). miRNAs are pervasive in animals and plants and act as posttranscriptional regulators that specifically guide target gene recognition to regulate the start or repression of gene transcription (Carthew and Sontheimer, 2009). Based on immunoprecipitation of c-Myc-tagged QDE-2 and isolation of its associated RNAs, Lee et al. (2010) discovered miRNA-like RNAs (milRNAs) in *Neurospora crassa*, with similar characteristics to miRNAs derived from animals and plants. milRNAs have since been identified in various fungal species, such as *Fusarium oxysporum* (Chen et al., 2014), *Sclerotinia sclerotiorum* (Zhou J. H. et al., 2012), *Metarhizium anisopliae* (Zhou Q. et al., 2012), and *Nosema ceranae* (Huang and Evans, 2016). They are produced by at least four diverse pathways that use a distinct combination of factors, including QDE-2, Dicers, the exonuclease QIP, and an RNase III domain-containing protein, MRPL3 (Lee et al., 2010). Compared to animals and plants, knowledge of miRNAs in fungi is somewhat limited, especially in bee fungal pathogens.

In our previous studies, the gut tissues of *A. apis*-inoculated and un-inoculated *Apis mellifera ligustica* worker larvae were prepared and subjected to small RNA-seq (sRNA-seq) and quality control of raw data, followed by investigation of differential expression patterns and the potential regulatory role of differentially expressed miRNAs (DEmiRNAs) in the infection process (Guo et al., 2019a,b), the results showed that in the early stage of *A. apis* stress (4-day-old larva), the *A. m. ligustica* worker larval DEmiRNAs miR-4331-y, miR-4968-y, miR-8440-y, novel-m0023-5p and novel-m0025-3p jointly regulate Wnt signaling pathway and endocytosis of host and can be used as potential molecular targets for chalkbrood control (Guo et al., 2019a), while during the late stage of *A. apis* stress, DEmiRNAs ame-miR-927b, miR-429-y and miR-8440-y may be involved in the regulation of serine protease and ubiquitin-mediated proteolysis (Guo et al., 2019b). Also, following deep sequencing of cDNA libraries, we previously identified 193 and 275 milRNAs in *A. apis* mycelium and spore samples, respectively (Chen et al., 2020). On basis of these obtained high-quality transcriptome datasets from *A. apis* spores and *A. apis*-infected larval guts (Guo et al., 2019a,b), milRNAs in *A. apis* invading *A. m. ligustica* worker larvae were characterized in the current work, followed by molecular validation of the expression and sequence of said milRNAs; additionally, DEmilRNAs were screened by comparative analysis and those DEmilRNAs within the significant profiles were further analyzed to explore the mechanism underlying fungal invasion; ultimately, a dual-luciferase reporter assay was conducted to verify the interaction between the DEmilRNAs and their target mRNAs. Our findings could lay the foundation for unveiling the mechanisms underlying the DEmilRNA-regulated pathogenesis of *A. apis*, offer a new insight into the interactions between *A. apis* and honey bee larvae, and provide promising candidates for further functional dissection and chalkbrood control.

## 2 Materials and methods

### 2.1 Fungi

The *A. apis* used in this work was deposited in China General Microbiological Culture Collection Center (CGMCC) under the microbiological culture collection number: 40895.

### 2.2 Source of sRNA-seq data from *Ascosphaera apis* spores

Following our previously established protocol (Guo et al., 2019b; Chen et al., 2020), *A. apis* stored at 4°C was transferred to PDA medium and cultured at 33 ± 0.5°C in a constant temperature and humidity chamber (Jingke, Shanghai, China). After 10 days of culturing, white mycelia were removed, and black fruiting bodies were harvested and transferred to an RNAase-free EP tube; 1 mL of sterile water was added to the EP tube, followed by complete grinding; the grinding fluid was centrifuged at 25°C, 7,000 × g for 3 min and the supernatant was then removed; 1 mL of sterile water was added, followed by centrifugation at 25°C, 7,000 × g for 3 min; the centrifugation was repeated twice to gain clean spores, which were then stored at 4°C until use. sRNA-seq of *A. apis* spores (AaCK group) was previously carried out according to the established protocol (Chen et al., 2020), followed by total RNA isolation, cDNA library construction, sRNA-seq, and strict quality control of raw data, with three biological replicates of the *A. apis* spore samples. The raw data were deposited in the NCBI Sequence Read Archive (SRA) database and linked to the BioProject number PRJNA560456.

### 2.3 Source of sRNA-seq data from *Ascosphaera apis*-inoculated *Apis mellifera Ligustica* larval guts

In another previous works (Guo et al., 2019a,b), the *A. m. ligustica* worker 3-day-old larvae (*n* = 9) in treatment group were fed 50 μL of artificial diet containing *A. apis* spores, with a final concentration of 1 × 10^7^ spores/mL; while the 3-day-old larvae (*n* = 9) in control group were fed 50 μL of artificial diet without fungal spores; the larvae in treatment and control groups were reared in two chambers under the conditions of 35.0 ± 0.5°C and 90% RH (relative humidity); the diets were replaced with new diets without spores every 24 h; the 4-, 5-, and 6-day-old larval guts were, respectively, dissected according to our established protocol (Guo et al., 2019c), nine gut tissue for each day-old; the prepared guts were quickly frozen in liquid nitrogen and then conserved at −80°C; the aforementioned experiments were repeated in triplicate; these gut samples were subjected to RNA isolation, cDNA library construction, and next-generation sequencing on the Illumina MiSeq™ system by Guangzhou Genedenovo Biotechnology Co., Ltd. (Ye et al., 2022). The raw data are available in the NCBI SRA database under the BioProject number PRJNA565629.

### 2.4 Identification of milRNAs in *Ascosphaera apis* during the infection process

Based on the sRNA-seq data from *A. apis-*inoculated *A. m. ligustica* larval guts mentioned above, milRNAs in *A. apis* invading the 4-, 5-, and 6-day-old larvae (AaT1, AaT2, and AaT3 groups) were identified following our previously described method (Xiong et al., 2020). Briefly, (1) clean tags from the sRNA-seq of the gut tissues of 4-, 5-, and 6-day-old *A. m. ligustica* workers were first mapped to the GenBank and Rfam databases to remove ribosomal RNA (rRNA), small cytoplasmic RNA (scRNA), small nucleolar RNA (snoRNA), small intranuclear RNA (snRNA), and transport RNA (tRNA) data; (2) unannotated clean tags were aligned to the *Apis mellifera* genome (assembly Amel 4.5) (Elsik et al., 2014) using the BLAST tool, and the mapped clean tags were then filtered; (3) unmapped clean tags were further aligned to the reference genome of *A. apis* (assembly AAP 1.0), and the mapped clean tags were deemed to be derived from *A. apis* during the infection process.

### 2.5 DEmilRNA screening and trend analysis

The miRNA expression level of each milRNA was estimated by transcript per million (TPM), which can avoid the effect of quantitative accuracy and normalized the expression level of sRNAs in different sequencing amounts. The formular was: TPM = T × 10^6^/N, in which T refers to the clean tags of miRNAs and N refers to the clean tags of total miRNAs. The data from the TPM normalization were used to identify the DEmilRNAs based on the number of genomic tags in each sample and compare the miRNA abundance among the three sets of libraries. Next, the expression levels of the milRNA in AaT1, AaT2, and AaT3 were normalized as log_2_(AaCK/AaT1), log_2_(AaCK/AaT2), and log_2_(AaCK/AaT3). Furthermore, trend analysis of the total DEmilRNAs was conducted using Short Time-series Expression Miner (STEM) software (Ernst and Bar-Joseph, 2006), with default parameters: Maximum unit change in model profiles between time points is 1, maximum output profile number is 20, and minimum ratio of fold change of DEmilRNA is no less than 2.0. Following the exported results from STEM software, those DEmilRNAs within significant profiles were used for the subsequent analyses.

### 2.6 Prediction, annotation, and enrichment analysis of target mRNAs

Following the method described by Fan et al. (2023), a combination of RNAhybrid (v2.1.2) + svm_light (v6.01) (Krüger and Rehmsmeier, 2006), Miranda (v3.3a) (Enright et al., 2003), and TargetScan (v7.0) (Allen et al., 2005) software was used to predict the target mRNAs of the DEmilRNAs, with default parameters set for each of the aforementioned software. Subsequently, the shared target mRNAs were considered the final targets with high confidence. Those DEmilRNAs and target mRNAs with binding free energy (△G) < −17 kcal/mol were selected for further investigation. Using the BLAST tool, the target mRNAs were, respectively, mapped to the GO (http://www.geneontology.org/, accessed on 5 November 2022) and KEGG databases (https://www.kegg.jp, accessed on 5 November 2022) to gain corresponding function and pathway annotations. By utilizing the tools on OmicShare platform KEGG, enrichment analysis, which adopts a hypergeometric test in terms of GO term or KEGG pathway to find out those significantly enriched in the targets compared to the whole background, was conducted. The *p*-value for each GO term or KEGG pathway enriched by targets was calculated using the formula:

$P=1-\sum_{i=0}^{m-1}\frac{\left(\begin{array}{c} M\\ i \end{array}\right)\left(\begin{array}{c} N-M\\ n-i \end{array}\right)}{\left(\begin{array}{c} N\\ n \end{array}\right)}$

where *N* is the number of all genes (background genes) with GO or KEGG annotations, n is the number of target genes in *N*, and m is the number of target genes annotated is the number of all genes annotated to a GO term or KEGG pathway, I is the number of target genes annotated to a GO term or KEGG. pathway.

### 2.7 Construction of DEmilRNA–mRNA sub-networks

As a fungal pathogen that exclusively infects honey bee larvae, *A. apis* has developed a specific mode of infection over a long period of time in a synergistic evolutionary process (Evison, 2015). When infecting insect hosts, the pathogenicity of fungal pathogens mainly depends on absorbing nutrients from the host to meet their own reproduction needs, as well as inhibiting and weakening the host natural immunity via diverse manners such as the secretion of chitinases, DNA methyltransferases, catalases, hexokinases, and various secondary metabolites (Wang et al., 2017; Qu and Wang, 2018). The MAPK cascade reaction has a special status among the fungal signaling pathways, which is closely associated with the virulence of fungal pathogens and assists the fungal infection process (Guo et al., 2020). In this study, we reviewed the previous studies on the biochemistry, molecular biology and histology of *A. apis* (Cornman et al., 2012), genes of MAPK cascades, secondary metabolite-related enzymes, and virulence factors were selected. Here, on basis of the annotations of the target mRNAs, those mRNAs relevant to virulence factors, secondary metabolite-related enzymes, secondary metabolites, and the MAPK signaling pathway were selected for construction of regulatory networks with corresponding DEmilRNAs, followed by visualization using Cytoscape software (Smoot et al., 2011), with △G < –20 kcal/mol.

### 2.8 Stem-loop RT-PCR and sanger validation of milRNAs

Six miRNAs (milR-3245-y, milR-10285-y, milR-1-y, milR-13-x, milR-33-x, and milR-375-y) were randomly selected for RT-PCR. Specific stem–loop primers and forward primers (F), as well as universal reverse primers, for each of these milRNAs were designed using Primer Premier 6 (Singh et al., 1998) (Supplementary Table 1) and then synthesized by Sangon Biotech Co., Ltd. (Shanghai, China). Using an RNA extraction kit (Promega, China), total RNA was extracted from the AaCK sample. Next, reverse transcription was performed with stem–loop primers, and the resulting cDNA was used as a template for PCR. On basis of the method described by Fan et al. (2023), PCR amplification was conducted and subjected to 1.8% agarose gel electrophoresis, followed by extraction of the expected fragments of milR-3245-y and milR-10285-y, ligation to the pESI-T vector (Yeasen, China), and transformation into *Escherichia coli* DH5*α*-competent cells. After microbial PCR, 1 mL of the bacterial solution with a positive signal was sent to Sangon Biotech Co., Ltd. (Shanghai, China) for single-end Sanger sequencing.

### 2.9 RT-qPCR detection of *Ascosphaera apis* DEmilRNAs and target mRNAs

A disintegrin and metalloproteinases (ADAMs) play a vital role in proteolysis, adhesion, signaling, and fusion (Meyer et al., 2010). Pyruvate kinase (PKIA) has been verified to catalyze the fungal glycolysis (Xu et al., 2020). Based on the result of trend analysis conducted in 2.5 section, both milR-6882-x and milR-7009-x were detected to continuously downregulated during the infection process of *A. apis*. Additionally, following target prediction performed in 2.6 section, milR-6882-x and milR-7009-x were observed to, respectively, target *ADM-B* and *PKIA*. Hence, RT-qPCR was carried out to determine the relative expression levels of milR-6882-x, *ADM-B*, milR-7009-x, and *PKIA*. On basis of the method described by Fan et al. (2023), the cDNA was synthesized for qPCR reaction, which was performed using the QuanStudio 3 Fluorescence quantitative PCR instrument (ABI, Los Angeles, CA, United States). The reaction system (20 μL) contained 10 μL of SYBR green dye, 1 μL of forward and reverse primers (2.5 μmol/L), 1 μL of cDNA template, and 7 μL of DEPC water. The reaction conditions were set as follows: 95°C pre-denaturation for 5 min; 95°C denaturation for 30 s; 60°C annealing and extension for 30 s; a total of 40 cycles for each group of the qPCR reaction, and the experiment was set to repeat three times. The *5.8S rRNA* (GenBank accession number: NR_178140) was used as the internal reference. The relative expression level of each DEmiRNA and target mRNA was calculated using the 2^−∆∆Ct^ method (Livak and Schmittgen, 2001). Each experiment was carried out with at least three samples in parallel and was repeated three times. The primers used in this work are detailed in Supplementary Table 1. A Student’s *t*-test was performed on the qPCR data using Graph Prism 8 software, and the significance of the differences and plots were calculated (ns: *p* > 0.05, *p < 0.05, **p < 0.01, ***p < 0.001, and ****p < 0.0001).

### 2.10 Dual-luciferase reporter assay

According to the method described by Lan et al. (2023) and Yuan et al. (2023), specific binding sites of the milR-6882-x identifying *ADM-B* 3′-UTR were acquired using the online software RNAhybrid (v2.1.2) (Krüger and Rehmsmeier, 2006). Subsequently, the milRNA binding sequences and matching mutant sequences of the target mRNAs were synthesized by Sangon Biotech Co., Ltd. (Shanghai, China) and cloned into XhoI and SacI sites of the pmirGLO reporter vector (Promega, China). This led to the construction of both an *ADM-B* 3′-UTR wild-type vector (ADM-WT) and a mutant-type vector (ADM-MUT), followed by plasmid extraction according to EasyPure^®^ HiPure Plasmid MiniPrep Kit protocol (Transgenbiotech, China). For high-transfection efficiency and low background expression of milR-6882-x, the mammalian HEK293T cell line was used for the luciferase reporter assay. The HEK293T cells were grown in DMEM/HIGH GLUCOSE medium (HyClone) containing 10% (vol/vol) heat-inactivated fetal bovine serum (FBS; Gibco, China) and 1 × antibiotic–antimycotic (Gibco, China) at 37°C under 5% CO_2_. For transfection, we seeded the HEK293T cells in 96-well plates and transfected them when they reached 80% confluence. PmirGLO-*ADM-B*-WT/MUT reporter vectors were co-transfected with milR-6882-x mimics or NC mimics into the HEK293T cells with Hieff Trans^®^ Liposomal Transfection Reagent (Yeasen, China). After 24–48 h of transfection, the cells were collected, lysed, and centrifuged for 10,000 rpm for 1 min. Then, 20 μL of the supernatant was taken and the firefly and Renilla luciferase activity was determined using a dual-luciferase reporter assay kit (Yeasen, China) with a dual-luciferase assay reporter system (Promega, China), according to the manufacturer’s instructions. The ratio of firefly luciferase/Renilla luciferase was calculated as the promoter activity. The same protocol used for the dual-luciferase reporter assay was applied to *PKIA* and milR-7009-x.

## 3 Results

### 3.1 Screening and quality control of sRNA-seq data from *Ascosphaera apis* during the infection process

On average, 8,644,606, 9,876,086, and 12,640,559 clean tags were identified from AaT1, AaT2, and AaT3 groups, respectively. Among these, 607,171, 1,241,615, and 1,944,927 clean tags were, respectively, mapped to the reference genome of *A. apis*, and the mapping ratios were 7.02, 12.57, and 15.39%, respectively (Table 1).

**Table 1:** Overview of the sRNA-seq data from *A. apis* spores and *A. apis* infecting *A. m. ligustica* worker larvae.

Group	Total clean tags	Mapped clean tags	Mapping ratio	Source
AaCK	9,888,848	7,080,369	71.60%	Chen et al. (2020)
AaT1	8,644,606	607,171	7.02%	This study
AaT2	9,876,086	1,241,615	12.57%	This study
AaT3	12,640,559	1,944,928	15.39%	This study

### 3.2 Identification, structural analysis, and molecular validation of *Ascosphaera apis* milRNAs

A total of 974 milRNAs, including 825 known and 149 new *A. apis* milRNAs, were identified. In detail, 380, 413, 485, and 511 milRNAs were identified from the AaCK, AaT1, AaT2, and AaT3 groups, respectively. There were 47 milRNAs shared by these four groups, with the number of unique ones being 266, 61, 91, and 112, respectively (Supplementary Figure 1).

**Figure 1:**
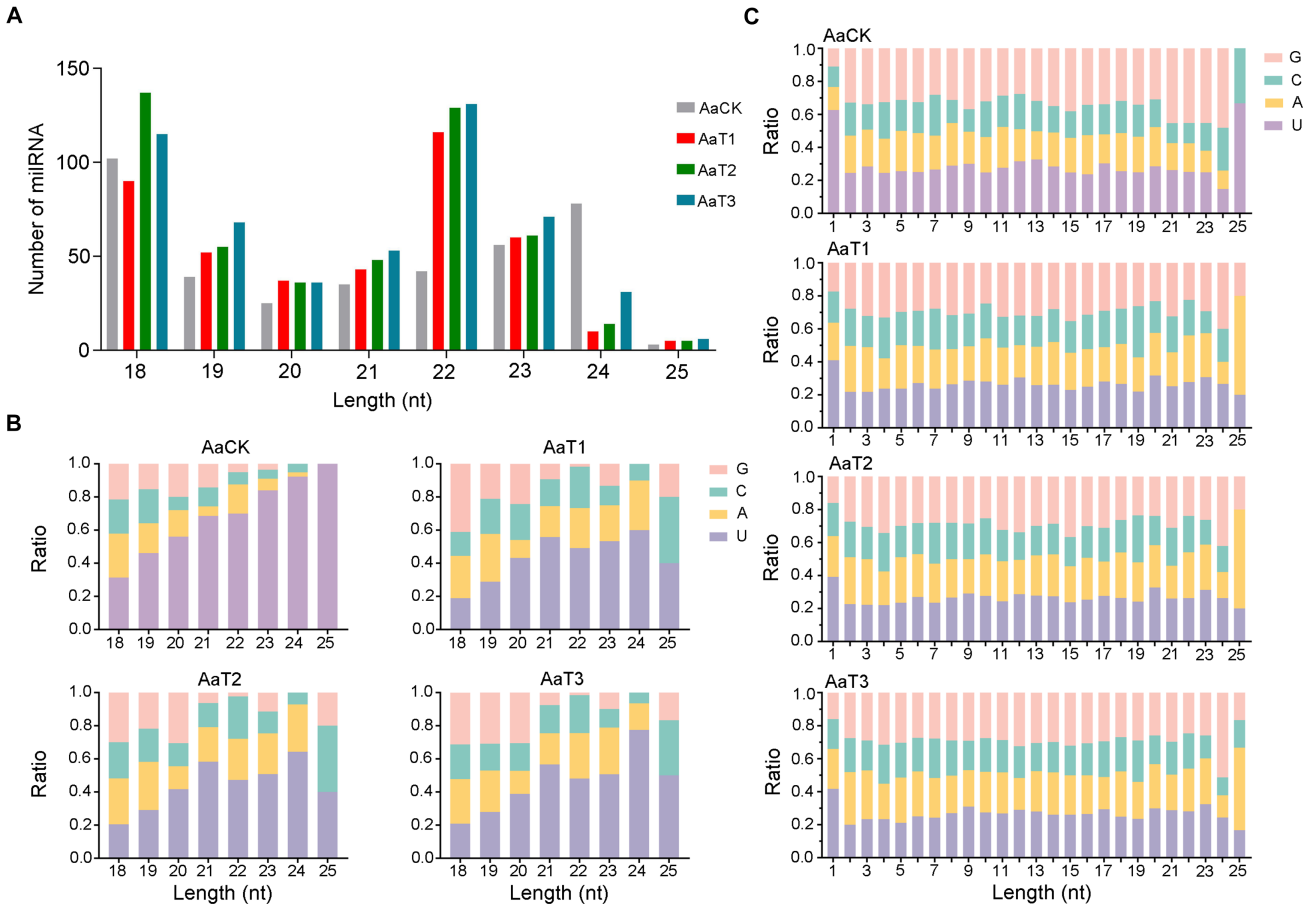
Structural characteristics of *A. apis* milRNAs. **(A)** Length distribution of the milRNAs; **(B)** First nucleotide bias of the milRNAs of different lengths; **(C)** Base bias of the milRNAs at each nucleotide.

In addition, structural analysis indicated that the length distribution of the milRNAs identified in the four groups were all between approximately 18 and 25 nt (Figure 1A); the most enriched lengths of the milRNAs in the AaT1, AaT2, and AaT3 groups were 18 and 22 nt, while the milRNAs in the AaCK group were mostly distributed at 18 nt, followed by 24 nt (Figure 1A). As shown in Figure 1B, the first base of the milRNAs of different lengths in the AaCK, AaT1, AaT2 and AaT3 groups was biased to U. Moreover, the base bias of the milRNAs at each nucleotide in these four groups varied (Figure 1C).

**Figure 2:**
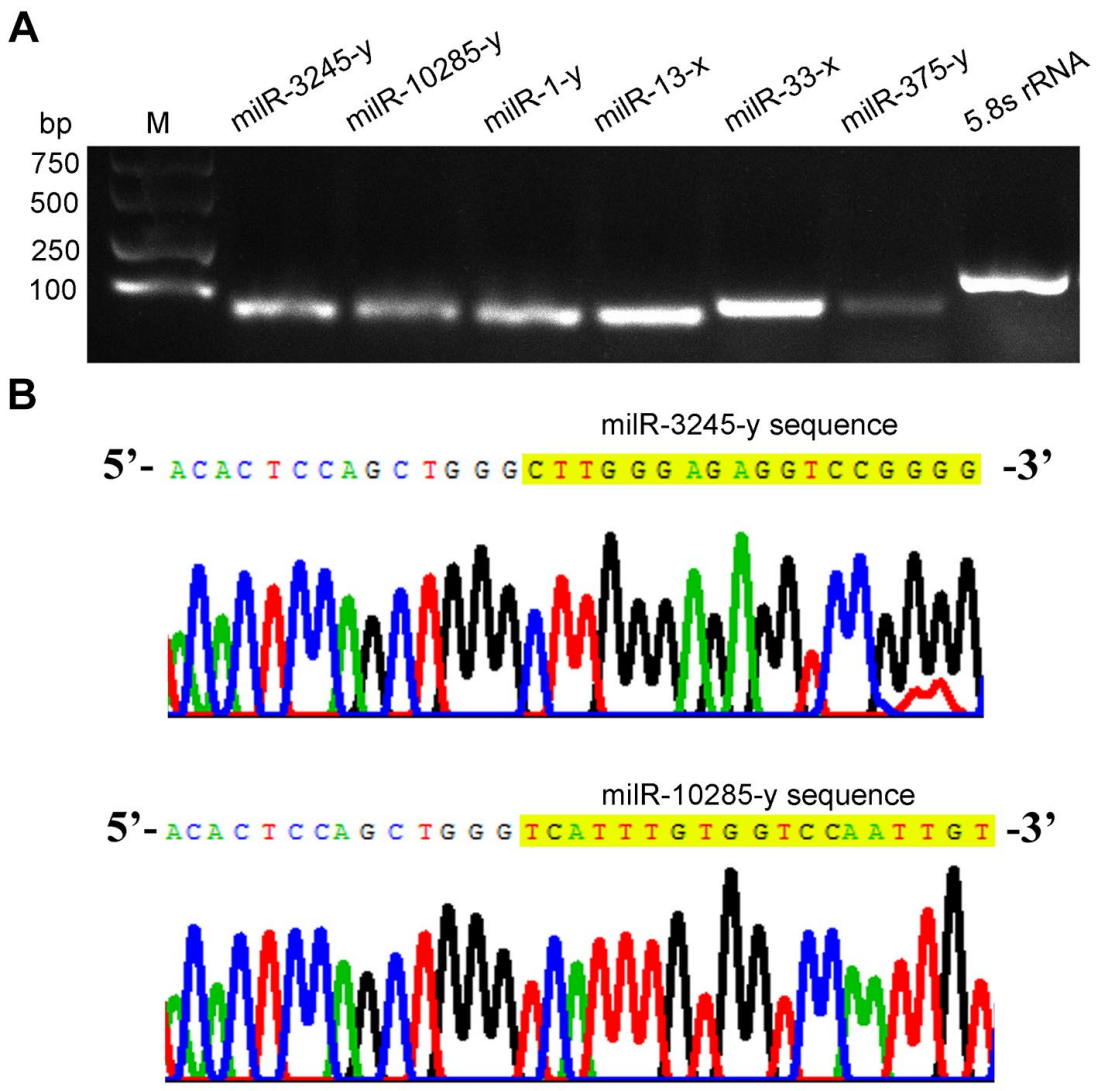
Molecular verification of *A. apis* milRNAs. **(A)** Agarose gel electrophoresis for the amplification products from stem–loop RT-PCR of six *A. apis* milRNAs and *5.8S rRNA*; **(B)** Sanger sequencing of the amplified products from milR-3245-y and milR-10285-y.

Further, stem–loop RT-PCR and agarose gel electrophoresis of six *A. apis* milRNAs were conducted, and the results suggested that the fragments of expected sizes (approximately 100 bp) were amplified from each of the abovementioned milRNAs (Figure 2A); the Sanger sequencing results confirmed the consistence between the actual and predicted sequences of milR-3245-y and milR-10285-y based on deep sequencing data (Figure 2B).

### 3.3 Trend analysis of *Ascosphaera apis* milRNAs

**Figure 3:**
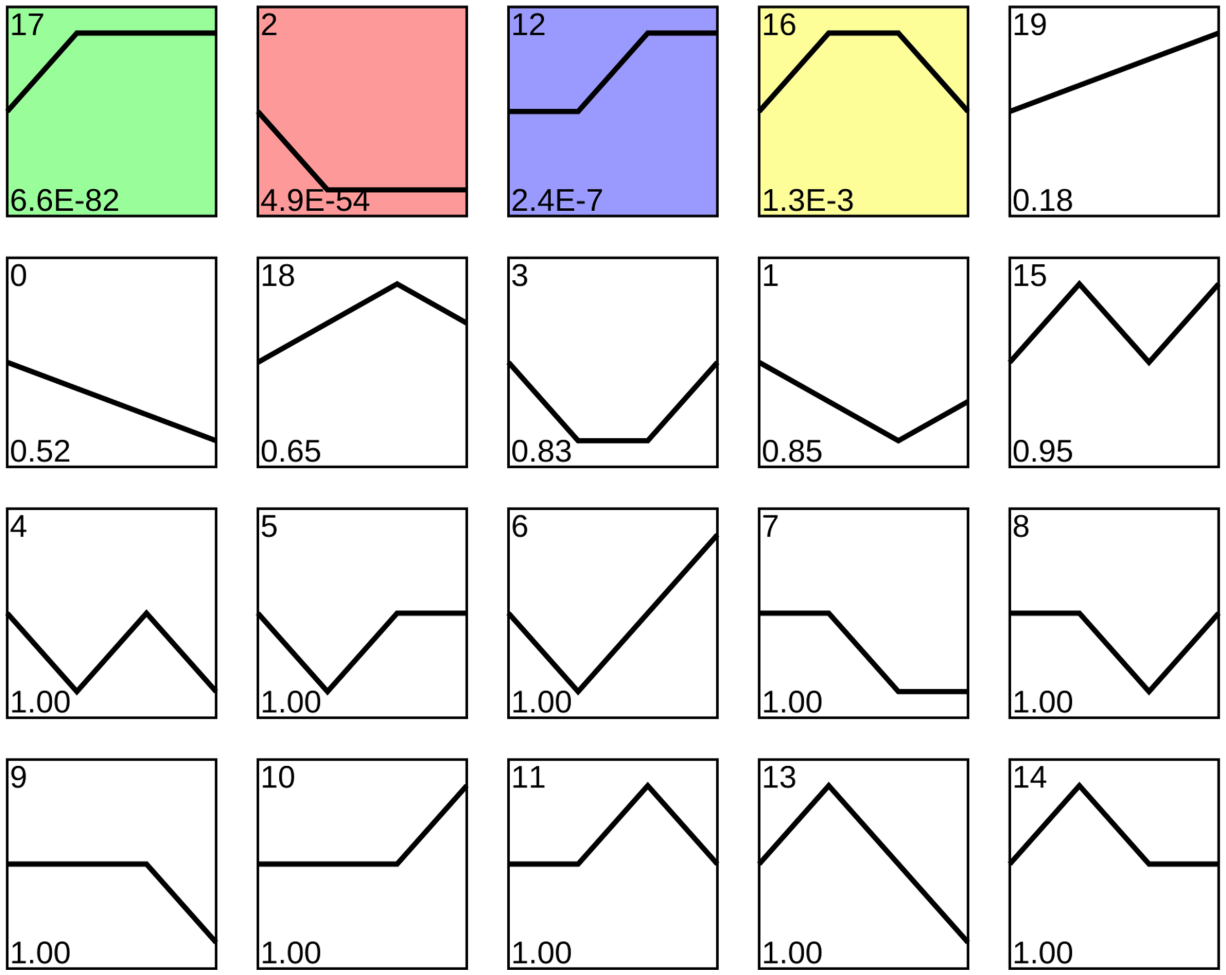
Four significant profiles identified by STEM analysis. Colorful squares represent significant trends, while Black-and-White ones represent non-significant trends; the numbers located in the upper left of each square indicate the quantity of milRNAs within each trend, whereas those located in the lower left of each square indicate the *p*-value of each trend.

Trend analysis indicated that 1,192 *A. apis* milRNAs were grouped into 20 profiles, including four significant profiles (profile2, profile12, profile17, and profile16), as shown in Figure 3. In detail, 348, 258, 35, and 27 milRNAs were included in profile2, profile17, profile12, and profile16, respectively. Targeting prediction suggested that 112, 35, 258, and 6 milRNAs within profile2, profile12, profile16, and profile17 could, respectively, target 120,031, 9,653, 8,758, and 82,999 mRNAs (Supplementary Table 2).

### 3.4 GO term and KEGG pathway analyses of the target mRNAs of the *Ascosphaera apis* DEmilRNAs within significant profiles

**Figure 4:**
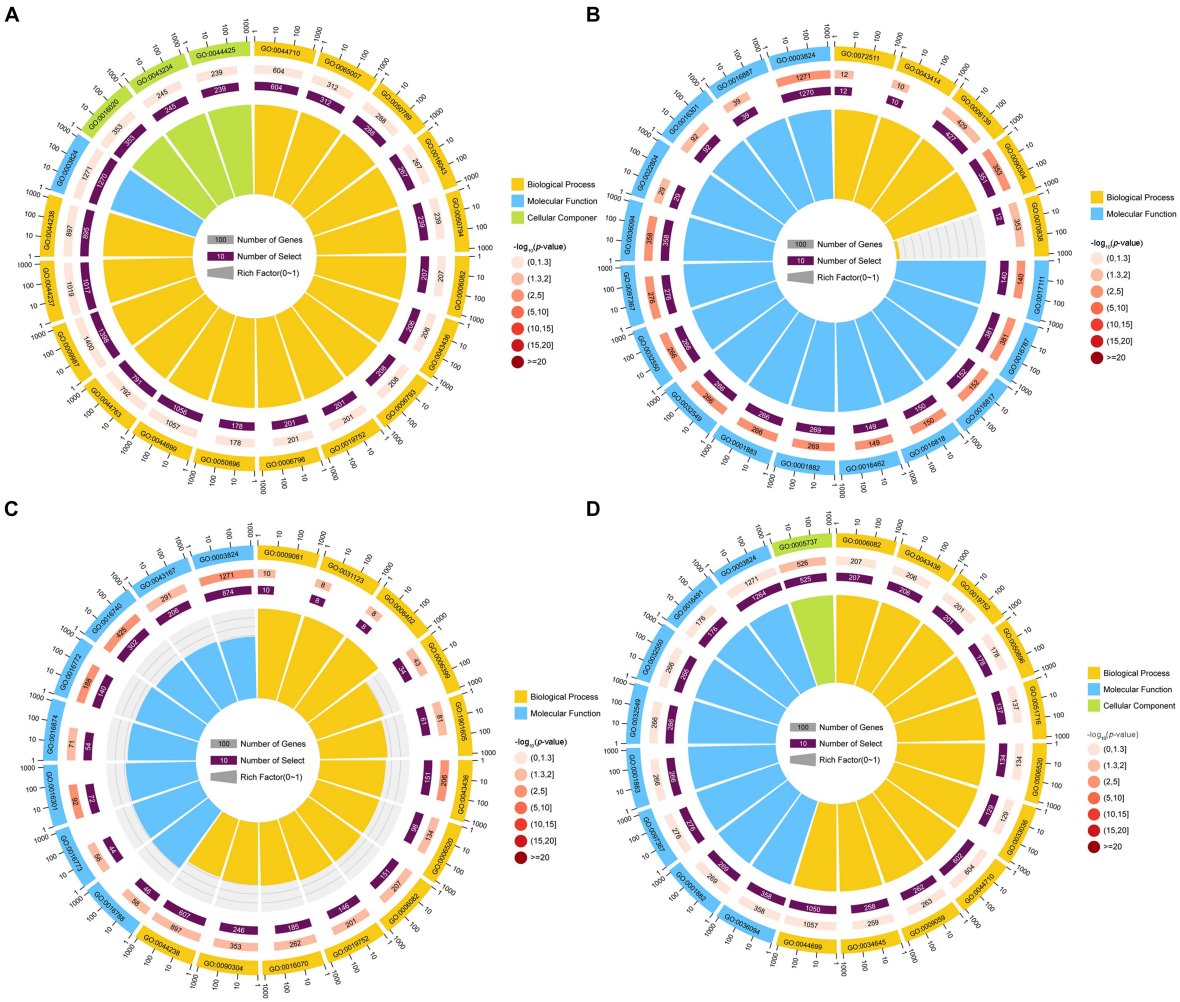
Top 20 GO terms significantly enriched by *A. apis* milRNA-targeted mRNAs. GO terms enriched by the target mRNAs of the *A. apis* milRNAs within profile2 **(A)**, profile12 **(B)**, profile16 **(C)**, and profile17 **(D)**.

GO term analysis showed that the target mRNAs of the DEmilRNAs within the four significant profiles were involved in 42 GO terms relevant to the cellular component, molecular function, and biological process, such as the cell process, metabolic process, single organize process, catalytic activity, binding, cell, and cell membrane; additionally, 26 GO terms were significantly enriched by target mRNAs of 35 milRNAs in profile12, including nucleoside-triphosphatase activity, hydrolase activity, hydrolase activity etc. (Figure 4B); 22 GO terms were significantly enriched by target mRNAs of 27 milRNAs in profile16, including catalytic activity, transferase activity, transferring phosphorus-containing groups, etc. (Figure 4C); one GO term (cytoplasm) was significantly enriched by target mRNAs of 27 milRNAs in profile17 (Figure 4D); while no GO term was found to be significantly enriched by target mRNAs of 348 milRNAs in profile2 (Figure 4A).

**Figure 5:**
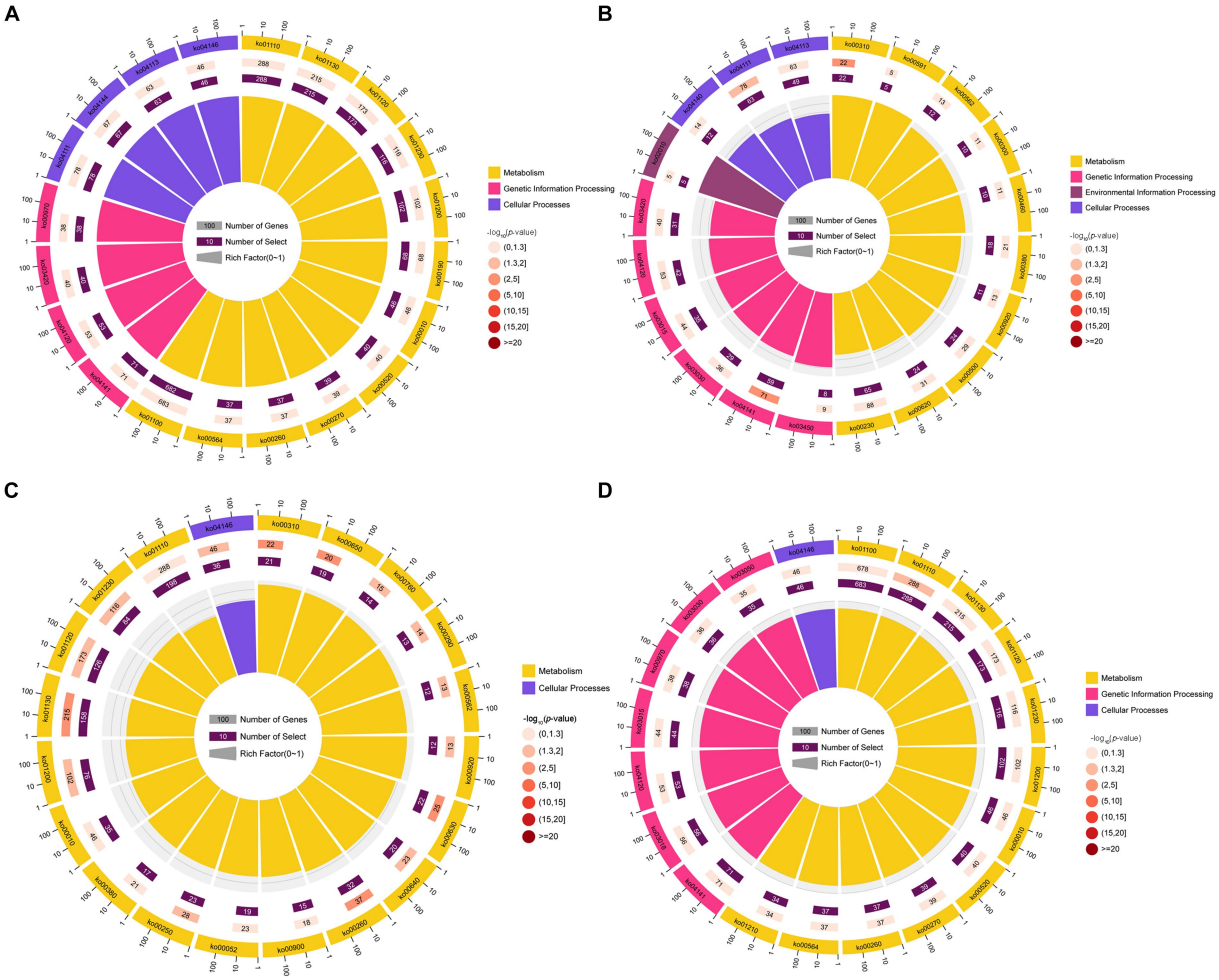
Top 20 KEGG pathways significantly enriched by *A. apis* milRNA-targeted mRNAs. Pathways enriched by the target mRNAs of the *A. apis* milRNAs within profile2 **(A)**, profile12 **(B)**, profile16 **(C)**, and profile17 **(D)**.

KEGG pathway analysis indicated that the target mRNAs of the DEmilRNAs within the four significant profiles were engaged in 120 KEGG pathways relative to metabolism, genetic information processing, environmental information processing, cellular processes, and organismal systems; additionally, the mRNAs targeted by the milRNAs in profile2 were significantly enriched in 120 pathways, such as metabolic pathways (ko01100) and biosynthesis of secondary metabolites (ko01110) (Figure 5A); the mRNAs targeted by the milRNAs in profile16 (upregulated and downregulated trend) were significantly enriched in 15 pathways such as lysine degradation (ko00310) and butanoate metabolism (ko00650) (Figure 5C); the target mRNAs of the milRNAs in profile12 and profile17 (upregulated trend) were enriched in 120 pathways, in which lysine degradation (ko00310), protein processing in endoplasmic reticulum (ko04141), cell cycle (ko04111), biosynthesis of secondary metabolites (ko01110) was significantly enriched (Figures 5B,D).

### 3.5 Investigation of the virulence factor-relevant sub-network between the *Ascosphaera apis* DEmilRNAs and targets

**Figure 6:**
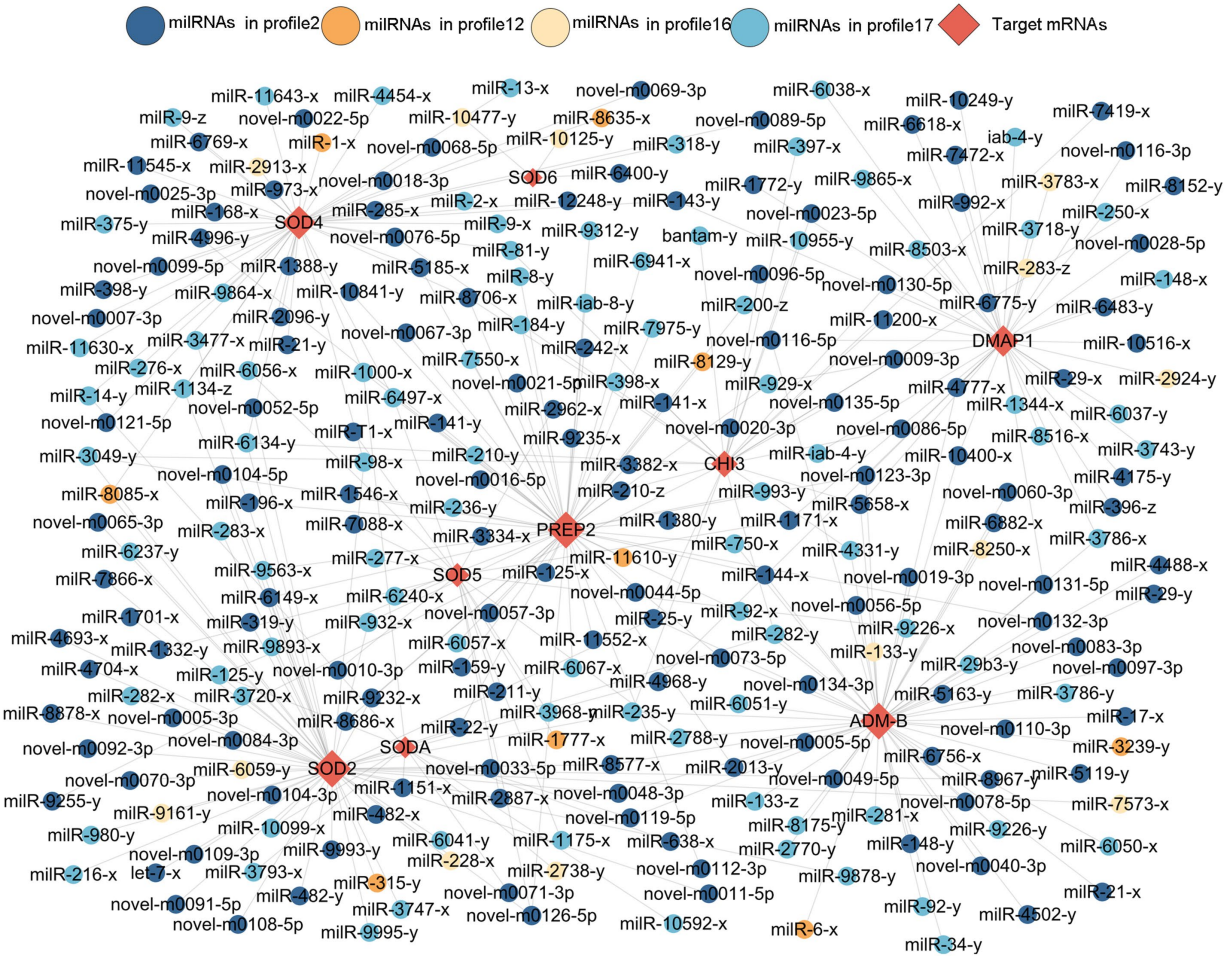
Sub-regulatory network between the *A. apis* DEmilRNAs in the four significant trends and targets associated with virulence factors.

Here, 258 DEmilRNAs showing significant trends were observed to target 10 genes encoding virulence factors (Figure 6; Supplementary Table 3). In detail, 145 milRNAs from profile2, 9 milRNAs from profile12, 13 milRNAs from profile16, and 91 milRNAs from profile17 were found to target six genes that encode superoxide dismutase, including *SODA*, *SOD*2, *SOD*4, *SOD*5, and *SOD*6, the metalloproteinase-encoding genes *ADM-B* and *PREP*2, the chitinase-encoding gene *CHI*3, and the DNA methyltransferase-encoding gene *DMAP*1. Additionally, 53 milRNAs targeted more than one gene concurrently, while 11 milRNAs (milR-25-y, milR-235-y, milR-1000-x, etc.) were able to target three genes and 42 milRNAs (milR-14-y, milR-22-y, milR-932-x, etc.) were found to target two genes.

### 3.6 Investigation of the sub-network associated with the secondary metabolite-related enzymes between the *Ascosphaera apis* DEmilRNAs and targets

A total of 299 DEmilRNAs were identified in the aforementioned four significant trends, which potentially targeted nine genes of encoding secondary metabolite-associated enzymes (Supplementary Figure 2; Supplementary Table 4). In detail, 162, 13, 13, and 111 milRNAs in profile2, profile12 profile16, and profile17 may, respectively, target three catalase-encoding genes (*CATB*, *CATA*, and *CTA*1), two genes encoding cysteine synthases (*CYS*12 and *CYSB*), the hexokinase-encoding gene *HXKA*, the pyruvate kinase-encoding gene *PKIA*, the tyrosinase-encoding gene *GRIF*, and the arginase-encoding gene *AGA*-1 (Supplementary Figure 2). Additionally, 83 milRNAs were capable of targeting more than one gene currently, while novel-m0011-3p and novel-m0081-3p were able to simultaneously target four genes. Furthermore, 13 milRNAs (milR-10048-y, milR-10516-x, milR-1344-x, etc.) were observed to target three genes, while 68 milRNAs, including bantam-y, milR-11200-x, and milR-1134-z, were able to target two genes.

### 3.7 Investigation of MAPK signaling pathway-related sub-network between *Ascosphaera apis* DEmilRNAs and targets

A total of 419 DEmilRNAs in the significant trends were observed to target nine genes related to the MAPK signaling pathway (Supplementary Figure 3; Supplementary Table 5). In detail, 216 milRNAs from profile2, 21 milRNAs from profile12, 13 milRNAs from profile16 and 163 milRNAs from profile17 targeted nine MAPK signaling pathway-related genes, namely, *STE*11, *MKH*1, *MKC*1, *STE*20*, STEA*, *WIS*4, *MKK*1, *STE*2, and *HOG*1. Additionally, 184 milRNAs targeted more than one gene concurrently, while milR-4561-y targeted seven genes, bantam-y targeted six genes, 18 milRNAs (milR-10125-y, milR-10249-y, milR-1151-x, etc.) targeted four genes, 53 milRNAs (let-7-x, milR-10592-x, milR-11545-x, etc.) targeted three genes, and 111 milRNAs (bantam-x, iab-4-x, milR-1000-y, etc.) targeted two genes.

### 3.8 Verification of binding relationships between the *Ascosphaera apis* DEmilRNAs and target genes

**Figure 7:**
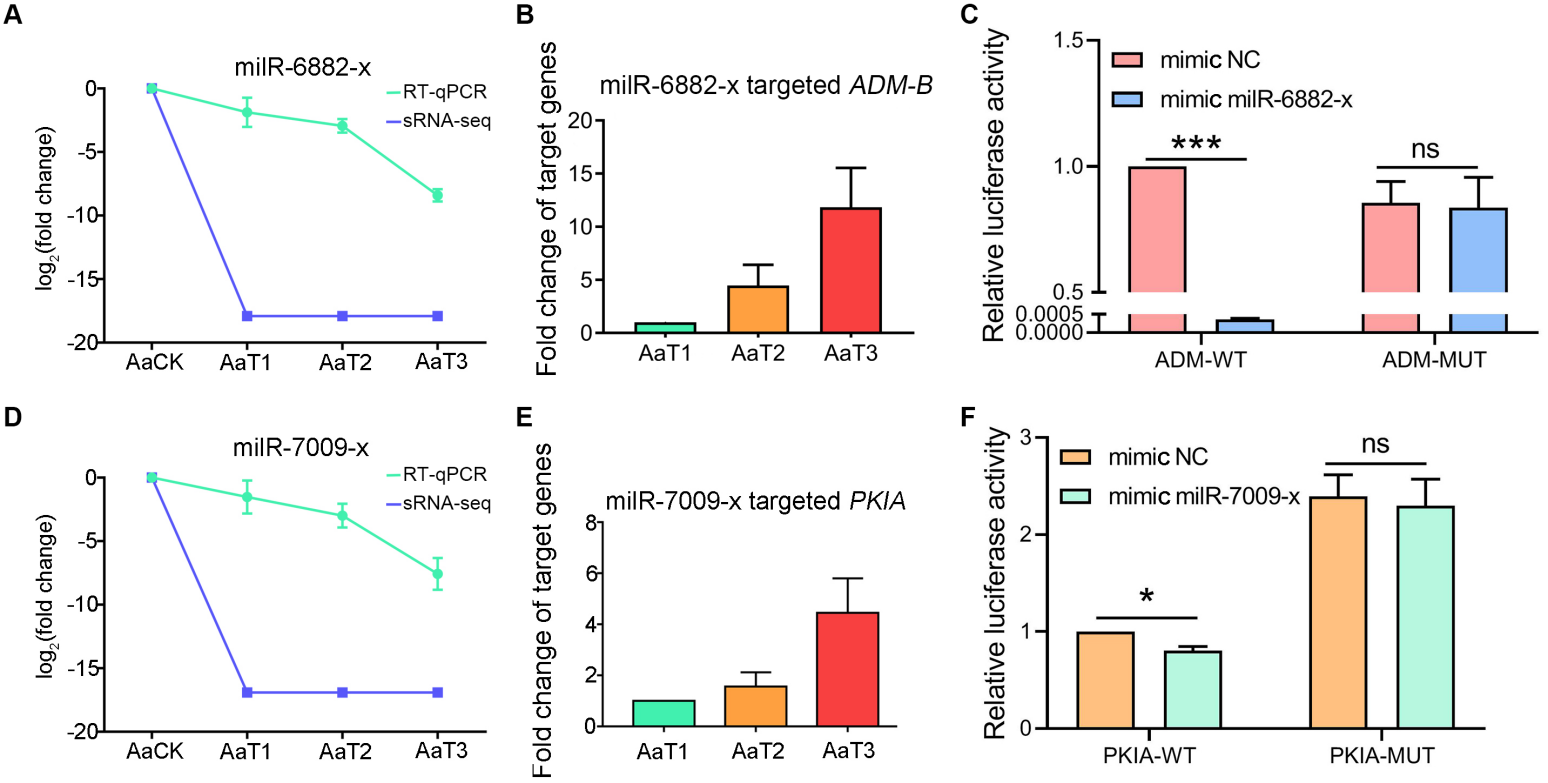
*A. apis* milRNAs target *ADM-B* and *PKIA*. **(A,D)** Relative expression level of milR-6882-x; **(B)** Relative expression level of *ADM-B*; **(C)** Dual-luciferase reporter assay of HEK293T cells co-transfected with milR-6882-x and pmirGLO vector containing target sequences of *ADM-B*; **(D)** Relative expression level of milR-7009-x; **(E)** Relative expression level of *PKIA*; **(F)** Dual-luciferase reporter assay of HEK293T cells co-transfected with milR-7009-x mimic and pmirGLO vector containing target sequences of *PKIA*.

RT-qPCR detection indicated that there was a negative relationship of the expression level between *ADM-B* and milR-6882-x (Figures 7A,B), as well as between *PKIA* and milR-7009-x (Figures 7D,E). In addition, the dual-luciferase reporter assay was suggestive of an interaction between *ADM-B* and milR-6882-x, as well as between *PKIA* and milR-7009-x (Figures 7C,F). Together, these results confirmed binding relationships between *ADM-B* and milR-6882-x and between *PKIA* and milR-7009-x.

## 4 Discussion

### 4.1 Resource identification initiative

In view of the pathogenesis of *A. apis* (Li et al., 2012), it is currently hard to completely isolate *A. apis* from honey bee larvae during the infection process. Following our previously established method, the transcriptome data from *A. apis* infecting *A. m. ligustica* worker larvae were filtered and combined with the sRNA-seq data from *A. apis* spores (Chen et al., 2020), based on which a total of 974 milRNAs were identified in this work. Since information about *A. apis* milRNAs is scarce, the identified 974 milRNAs enrich the reservoir of *A. apis* milRNAs and offer a valuable resource for further functional investigation.

Here, the length distribution of the identified milRNAs in the AaCK, AaT1, AaT2, and AaT3 groups were observed to range from 18 to 25 nt, the first base was biased to U, and the base bias of the milRNAs at each nucleotide was different (Figure 1B). The structural features of the *A. apis* milRNAs are analogous to those of other fungi, such as *S. sclerotiorum* (Zhou J. H. et al., 2012), *M. anisopliae* (Zhou Q. et al., 2012), and *Ganoderma lucidum* (Chen et al., 2012), indicative of the conservatism of milRNAs in various fungal species. Further analysis showed that the most enriched lengths of milRNAs in the AaCK group were distributed at 18 nt followed by 24 nt, whereas the milRNAs in the AaT1, AaT2, and AaT3 groups were mostly distributed at 18 and 22 nt (Figure 1A). This difference reflects the alteration of the length distribution of the *A. apis* milRNAs before and after infection of *A. m. ligustica* worker larvae. This is similar to the finding from tomato, in which the length of the sRNAs was changed after inoculation with *Pochonia chlamydosporia* (Pentimone et al., 2018). The results suggest that the length change of *A. apis* milRNAs may be involved in the fungal pathogenic process.

Fungal milRNAs are involved in cellular activity, material metabolism, and signal transduction through the regulation of target genes, thus playing a crucial role in growth, development, reproduction, and proliferation (Liu et al., 2016). The STEM clustering method applies a considerable number of genes and a limited number of time points to identify expression profiles that exhibit statistically significant temporal patterns, along with the genes associated with these profiles (Ernst and Bar-Joseph, 2006). In this work, trend analysis was performed and 668 milRNAs in four significant profiles were discovered (Figure 3). Target mRNAs of the milRNAs in profile2, profile12, profile16, and profile17 were observed to be significantly enriched in a series of functional terms of great importance, including the cell process, metabolic process, single organize process, catalytic activity (Figure 4). In addition, these targets were involved in an array of pathways such as MAPK signaling pathways, endocytosis, biosynthesis of secondary metabolites, cell cycle, and glutamate metabolism (Figure 5). The results demonstrated that the *A. apis* milRNAs in the four significant profiles likely participated in modulating diverse functional terms and pathways during the infection of *A. m. ligustica* worker larvae.

**Figure 8:**
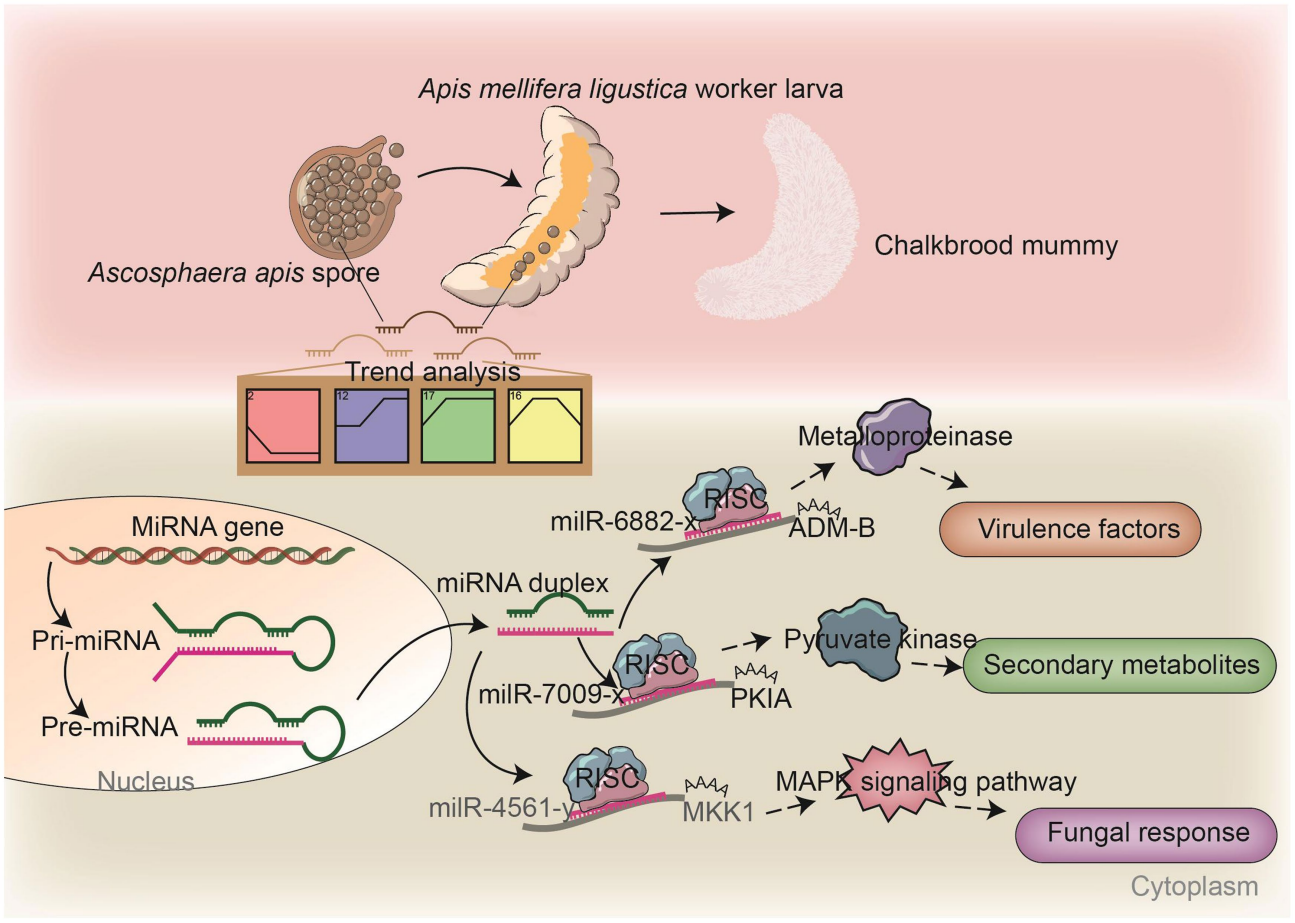
A hypothetical working model of the DEmilRNA-mediated regulation in *A. apis* invading *A. m. ligustica* worker larvae.

Insect fungal pathogens have evolved in synergy with their hosts over a long period of time; their pathogenicity relies on not only the exploitation of host nutrition to satisfy their own reproduction, but also on the suppression and weakening of insects’ natural immunity, such as the secretion of chitinases and DNA methyltransferases in combination with endocytosis to suppress the host immune response (Qu and Wang, 2018). Chitinase is involved in the synthesis of fungal cell walls and plays a key role in maintaining the cell morphology, stress resistance, and pathogenicity of fungi (Fiorin et al., 2018). In this current work, we observed that 17 milRNAs (miR-141-x, miR-7419-x, miR-9255-y, etc.) from profile2 and nine milRNAs (bantam-y, miR-282-y, miR-4331-y, etc.) from profile17 could jointly target the mRNAs of the *CHI*3 gene (KZZ95064.1) (Figure 6). This indicates that the corresponding DEmilRNAs are likely to regulate the pathogenesis of *A. apis* by modulating the expression of chitinase-encoding genes. ADAMs showed four main functions: proteolysis, adhesion, signaling, and fusion (Black et al., 1997; Seals and Courtneidge, 2003; Blobel, 2005; Mitsui et al., 2006) those were present in a number of fungi including *Cryptococcus neoformans* and *Magnaporthe grisea*. Demonstration of proteolytic activity for *ADM-B* indicates a possible sheddase role for fungal ADAMs (Lavens et al., 2005). In this study, milR-6882-x was suggested to target *ADM-B* and their expression levels were negatively related (Figures 7A–C), implying that milR-6882-x was likely to regulate the proteolysis, adhesion, signaling, and fusion of *A. apis* during the infection of *A. m. ligustica* worker larvae through interaction with the *ADM-B* gene (Figure 8). Accumulating evidence suggests that DNA methyltransferases play a vital role in the growth and virulence of fungal pathogens (Jeon et al., 2015; Wang et al., 2017). Wang et al. (2017) discovered that knockdown of *MrDIM*-2 in *Metarhizium robertsii* results in the downregulated expression of several genes encoding cysteine proteases, followed by the reduction of spore activity and pathogenicity. In *Magnaporthe oryzae*, knockout of the DNMTase-encoding gene *DIM-*2 has given rise to abnormalities of colony morphology, radial growth, and spore formation processes, ultimately leading to a significant reduction of fungal pathogenicity (Jeon et al., 2015). In a previous work, we detected that six upregulated and seven downregulated milRNAs in the gut of 6-day-old *Apis cerana cerana* worker larvae infected by *A. apis* could collectively target *DMAP*1 (KZZ94679.1), a gene encoding DNA methyltransferase (Xiong et al., 2020). In this study, 41 DEmilRNAs from profile2, profile16, and profile17 were observed to jointly target the mRNAs of *DMAP*1 (KZZ94679.1), indicative of the regulation of DNA methylation via the DEmilRNA–*DMAP*1 axis during the fungal infection process. Together, these results are suggestive of complex interactions between *A. apis* and the larvae of two different honey bee species mentioned above. *DMAP*1 may be a promising molecular target for the control of chalkbrood disease. In the near future, functional dissection of both DEmilRNAs and *DMAP*1 will be conducted based on our previously established platforms (Guo et al., 2022; Wu et al., 2023).

In fungi, secondary metabolites play a critical part in reproductive development, morphological differentiation, spore germination, and interactions with other organisms (Calvo et al., 2002; Keller, 2019). *Paenibacillus larvae*, a Gram-positive bacterium capable of infecting *A. mellifera* larvae and causing American foulbrood, is able to establish absolute dominance in the competition for host nutrients by synthesizing and secreting non-ribosomal peptides and peptide–polyketide mixtures to inhibit other bacteria and fungi during the infection process (Müller et al., 2015). As a widespread fungal pathogen for insects, *Beauveria bassiana* secretes secondary metabolites that are highly pathogenic during the invasion process, which can also toxify the host carcass to limit competition for host nutrients by other microorganisms (Fan et al., 2017). In the present study, we found that the target mRNAs of *A. apis* DEmilRNAs are associated with a suite of secondary metabolites, such as hexokinase, catalase, cysteine synthase, pyruvate kinase, tyrosinase, and arginase (Supplementary Figure 2). Previous studies have shown that hexokinase, a key protein involved in the glucose phosphorylation, which is a pathway widely present in fungi, is closely related to intracellular glycolysis, cellular autophagy, fungal pathogenicity (Rui and Hahn, 2007), and the formation of secondary metabolites. Herein, we found that 114 milRNAs (milR-203-y, milR-67-y, milR-9878-y, etc.) potentially targeted the mRNAs of three hexokinase genes (KZZ91574.1, KZZ89317.1, and KZZ97164.1), such as milR-305-x in profile17 with an upregulated trend that can target the mRNAs of *HXKA* (KZZ89317.1) and milR-122-x in profile2 with a downregulated trend that can target the mRNAs of *HXKA* (KZZ97164.1) (Supplementary Figure 2). In summary, these results suggest that *A. apis* may facilitate its proliferation by regulating secondary metabolites in a DEmilRNA-dependent manner. Meanwhile, *A. m. ligustica* worker larvae might defend against invasion by *A. apis* via DEmilRNA-mediated regulation of secondary metabolites, reflecting the complexity of host–pathogen interactions. Typically, fungal glycolysis is a sequence of reactions catalyzed by ten enzyme such as *PKIA* and phosphofructokinase (PFK) (Chroumpi et al., 2020). In order to survive in their niches, fungi have to be able to accommodate their metabolism to the energy source available. Glycolysis is one of the major pathways of central metabolism and plays a key role in the growth of almost all organisms (Elsik et al., 2014). It converts glucose into pyruvate along with the formation of adenosine triphosphate (ATP) and nicotinamide adenine dinucleotide (NADH). Here, dual luciferase assay verified the targeting relationship between milR-7009-x and *PKIA* (Figures 7D–F). Hypothetically, milR-7009-x modulated the proliferation and infection of *A. apis* via modulation of the by regulating the expression of pyruvate kinase encoding gene *PKIA*.

Pathogenic fungi are able to percept extracellular information and respond to stimuli from the external environment through a complex signaling cascade composed of Ca^2+^, cyclic adenosine monophosphate (cAMP), and mitogen-activated protein kinase (MAPK) (Jiang et al., 2018). The MAPK cascade is a pivotal signaling pathway involved in the regulation of fungal mating and reproduction, osmotic pressure regulation, spore formation, and virulence, which depends on MAP kinases that enhance fungal invasion of their hosts (Turrà et al., 2014). The cAMP signaling pathway is involved in fungal attachment and cell formation and plays a role in mycelial tip growth, carbon metabolism, and pathogenicity (D’Souza and Heitman, 2001). Our team previously observed that the expression of numerous genes related to the MAPK signaling pathway in *A. apis* infecting *A. c. cerana* and *A. m. ligustica* worker larvae was induced and activated (Guo et al., 2017). During the fungal infection of 6-day-old *A. c. cerana* worker larvae, 114 milRNAs (bantam-y, milR-2-x, milR-3-x, etc.) were upregulated and 88 milRNAs (milR-29-x, milR-319-y, milR-4968-y etc.) downregulated, and these milRNAs potentially target genes such as MAP kinase gene *WIS*4 (KZZ97981.1), pheromone mating factor gene *STE*2 (KZZ87749.1), and serine/threonine protein kinase gene *STE*20 (KZZ87358.1) in *A. apis* (Xiong et al., 2020). Here, it is noted that 413 DEmilRNAs potentially target nine mRNAs of the genes enriched in the MAPK signaling pathway; among these, as many as 117 DEmilRNAs potentially target the mRNAs of the mitogen-activated protein kinase gene *HOG*1. This indicates that the MAPK signaling pathway in *A. apis* was dynamically modulated by the corresponding DEmilRNAs, thereby affecting fungal adaptation and proliferation in the larval guts (Supplementary Figure 3). MAP kinases, which are dependent on MAPK, can help pathogenic fungi to infect hosts. Here, milR-4561-y was detected to target *MKK*1, which participated in the regulation of several critical processes such as conidiation, multi-stress tolerance, and pathogenicity (Xie et al., 2021). We speculated that milR-4561-y was involved in the modulation of the pathogenisis of *A. apis* by controlling the *MKK*1 gene expression (Figure 8). Currently, the *Agrobacterium tumefaciens*-mediated fungal genetic transformation (ATMT) system has been successfully applied for functional investigation of genes in various entomopathogenic fungi, including *Fusarium oxysporum* and *Aspergillus flavus* (Mullins et al., 2001; Leclerque et al., 2004; Yuan et al., 2018). One of our future directions is to establish the ATMT system of *A. apis* and further explore the function of key DEmilRNAs (milR-7009-x, milR-6882-x, milR-4561-y, etc.) in *A. apis* during the infection process.

In conclusion, a total of 974 milRNAs were identified in *A. apis*, with a length distribution between approximately 18 and 25 nt and a similar base bias to other fungi. Four significant trends including 669 DEmilRNAs were discovered in *A. apis* infecting *A. m. ligustica* worker larvae. The target mRNAs of these DEmilRNAs were engaged in 42 functional terms and 120 pathways, and the corresponding DEmilRNAs, such as milR-6882-x, milR-7009-x, milR-305-x, milR-122-x, and bantam-y, are likely to modulate the pathogenesis of *A. apis* during the infection process through the modulation of the expression of the target genes involved in secondary metabolites, virulence factors, and the MAPK signaling pathway.

## Data availability statement

The datasets presented in this study can be found in online repositories. The names of the repository/repositories and accession number(s) can be found in the article/Supplementary material.

## Author contributions

XF: Data curation, Formal analysis, Methodology, Software, Validation, Writing – original draft. XG: Data curation, Formal analysis, Methodology, Software, Validation, Writing – original draft. HZ: Data curation, Software, Writing – original draft. ZL: Data curation, Software, Writing – original draft. XJ: Validation, Writing – original draft. XL: Validation, Writing – original draft. SG: Validation, Writing – original draft. HJ: Validation, Writing – original draft. YW: Visualization, Writing – original draft. ZH: Visualization, Writing – original draft. DC: Conceptualization, Funding acquisition, Project administration, Writing – review & editing. RG: Conceptualization, Funding acquisition, Project administration, Supervision, Writing – review & editing.
