# Supplementary figures and images for: Transcriptional dynamics and regulatory function of milRNAs in Ascosphaera apis invading Apis mellifera larvae
Source: Front Microbiol. 2024 Apr 8;15:1355035. doi: 10.3389/fmicb.2024.1355035 (PMC11033319; doi:10.3389/fmicb.2024.1355035)

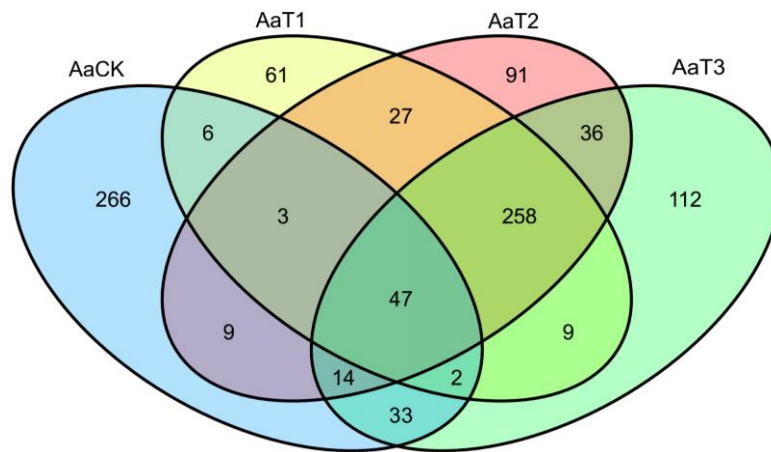

**Supplementary Fig. 1.** Venn diagram of the *A. apis* milRNAs discovered in the four groups.

Supplement: Supplementary file 6 [file Data_Sheet_1.PDF]

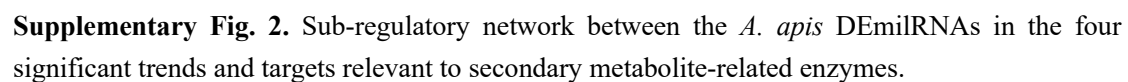

Supplement: Supplementary file 7 [file Data_Sheet_2.PDF]

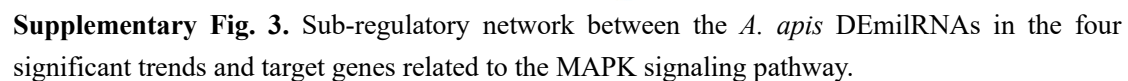

Supplement: Supplementary file 8 [file Data_Sheet_3.PDF]
